# Optimierung der Arbeit von Gedächtnisambulanzen unter Gesichtspunkten von „value-based healthcare“ – ein Ansatz aus dem Zentrum für Gedächtnisstörungen der Uniklinik Köln

**DOI:** 10.1007/s00115-025-01903-w

**Published:** 2025-11-17

**Authors:** Anna Schönberger, Ann-Katrin Schild, Annika Steinmetz, Franziska Simandi, Gloria S. Benson, Lucrezia Hausner, Michael Schöttler, Bastian Hennig, Arne Knudsen, Franziska Maier, Lutz Frölich, Frank Jessen

**Affiliations:** 1 Klinik und Poliklinik für Psychiatrie und Psychotherapie, Medizinische Fakultät, Uniklinik Köln, Straße 62, 50937 Köln, Deutschland; 2 Zentralinstitut für Seelische Gesundheit, Mannheim, Deutschland; 3 Deutsches Netzwerk Gedächtnisambulanzen (DNG), https://deutschesnetzwerkgedachtnisambulanzen.clubdesk.com/; 4 Roche Pharma AG, Grenzach-Wyhlen, Deutschland; 5 Roche Diagnostics Deutschland GmbH, Mannheim, Deutschland; 6 Deutsches Zentrum für neurodegenerative Erkrankungen (DZNE), Köln/Bonn, Deutschland

**Keywords:** Demenz, Alzheimer-Krankheit, Diagnostik, Gesundheitsökonomie, Patientenverorgung, Dementia, Alzheimer’s disease, Diagnostics, Health economics, Patient care

## Abstract

**Hintergrund:**

Gedächtnisambulanzen in Deutschland stehen aufgrund zunehmender Patientenzahlen und erster verfügbarer krankheitsmodifizierender Therapien für die Alzheimer-Krankheit vor großen Herausforderungen. Es müssen Kapazitäten für Beratung, biomarkerbasierte Diagnostik, Therapien und Kontrolluntersuchungen geschaffen werden, sodass eine Anpassung der Arbeitsabläufe notwendig ist. „Value-based healthcare“ (VBHC) bietet die Chance, Optimierungen so zu gestalten, dass der patientenbezogene Nutzen neben Kosten- und Effizienzgesichtspunkten im Mittelpunkt steht.

**Ziel der Arbeit:**

Dieses Projekt wendet VBHC erstmals auf Gedächtnisambulanzen an, um nach seiner zentralen Formel („value = outcome/costs“) ein besseres Outcome für Patienten und Angehörige bei einem effizienteren Einsatz bestehender Ressourcen („costs“) zu erreichen.

**Methoden:**

Durch eine erste Befragung von Patienten und Angehörigen wurden wesentliche Aspekte in Bezug auf VBHC erfasst und hierauf basierend bestehende Arbeitsabläufe modifiziert. Diese Modifikationen wurden durch eine zweite Befragung und Analysen vor allem prozessorientierter Aspekte evaluiert.

**Ergebnisse:**

Bereits bei der ersten Befragung bestand eine generelle Zufriedenheit mit der Vorstellung in der Gedächtnisambulanz. Als zentraler Kritikpunkt erwies sich aber die Dauer des Diagnostikprozesses. Nach Modifikation konnten Dauer und Umfang der Diagnostik reduziert werde. Die Befragten bewerteten die modifizierten Abläufe besser und Ressourcen wurden eingespart.

**Diskussion:**

In unserer Ambulanz wurde durch eine erhöhte Behandlungszufriedenheit („outcome“) bei verringerten Personalbindungszeiten („costs“) eine Verbesserung im Sinne von VBHC erzielt. Dieses Modell kann anderen Gedächtnisambulanzen als Vorbild für die Entwicklung einer patientenzentrierteren und effizienteren Versorgung dienen.

## Abstract (en)

**Background:**

Memory clinics in Germany are facing major challenges due to increasing numbers of patients and the first available disease-modifying treatments for Alzheimer’s disease. Capacities for counselling, biomarker-based diagnostics, drug administration and follow-up examinations must be achieved, which creates the need for modified workflows. Value-based healthcare (VBHC) aims at optimizing the value for patients (outcome in relation to costs) and can serve as a framework for a patient-oriented increase in efficacy.

**Objective:**

This project applied approaches of VBHC to analyze and improve the diagnostic processes in our memory clinic in order to achieve a better value for patients and care partners with a more efficient use of existing resources.

**Methods:**

In a first survey among memory clinic patients and relatives the essential aspects in relation to VBHC were collated and based on the results the existing workflow processes were modified. These modifications were evaluated by a second survey and analysis particularly of process-oriented aspects.

**Results:**

The first survey revealed a general satisfaction with the presentation in the memory clinic. The main point of criticism was the duration of the diagnostic process. After the modification the duration and extent of the diagnostics could be reduced. The second evaluation showed improved patient and care partner satisfaction. The respondents considered the modified trajectories to be better and resources were conserved.

**Conclusion:**

In our memory clinic an improvement in the sense of VBHC could be achieved through an increased satisfaction with the treatment (outcome) and reduced personnel binding times (costs). This approach can serve as a model for other memory clinics for the development of a more efficient and patient-centered care.

## Hintergrund

Gedächtnisambulanzen fokussieren auf die Früh- und ätiologische Differenzialdiagnostik von Demenzerkrankungen sowie auf Beratung und therapeutische Maßnahmen [8]. Sie orientieren sich dabei an der S3-Leitlinie Demenzen [23]. Für die Alzheimer-Krankheit wurden in der jüngsten Vergangenheit monoklonale Antikörper entwickelt, die erstmalig spezifisch gegen an der Pathogenese beteiligte Proteine gerichtet sind und das Fortschreiten der Symptomatik verlangsamen [3, 12]. Die Antikörper Lecanemab und Donanemab sind in vielen Ländern zugelassen und dort bereits Teil der Regelversorgung [10]. In der Europäischen Union erfolgte die Zulassung von Lecanemab und Donanemab für die Behandlung der leichten kognitiven Störung und der leichtgradigen Demenz bei Alzheimer-Krankheit im Jahr 2025 [24]. Darüber hinaus sind derzeit zahlreiche weitere krankheitsmodifizierende Therapien in der klinischen Erprobung und es ist von weiteren Zulassungen in den kommenden Jahren auszugehen [1]. Im Bereich der Diagnostik werden blutbasierte Biomarker in die Versorgung kommen, die Liquordiagnostik oder Positronenemissionstomographie in Teilen ersetzen werden [20]. Diese grundlegenden Innovationen werden zusammen mit der demographischen Alterung der Gesellschaft [7] zu einem starken Anstieg der Patientenzahlen in Gedächtnisambulanzen führen, was eine Herausforderung darstellt, da Kapazitäten begrenzt sind und Wartezeiten schon heute oft mehrere Monate betragen. In Bezug auf die neuen Therapien müssen zusätzliche Ressourcen geschaffen werden, um intravenöse Gaben sowie die geforderten Kontrolluntersuchungen mittels Magnetresonanztomographie (MRT) zu ermöglichen. Eine Anpassung der derzeitigen Abläufe ist daher erforderlich. Vorhandene Ressourcen müssen effizient und so eingesetzt werden, dass sie den größtmöglichen Nutzen für Patienten erbringen.

Vor diesem Hintergrund ist das durch den Ökonom Michael Porter entwickelte Konzept „value-based healthcare“ (VBHC; [15]) von besonderer Relevanz [4, 19]. Porters Hauptkritikpunkt am Gesundheitssystem ist, dass sich viele Akteure über die Anzahl der durchgeführten Untersuchungen und Behandlungen finanzieren, sodass ein Anreiz besteht, möglichst viele Leistungen zu erbringen. Der Nutzen dieser Maßnahmen für die Patienten steht hingegen nicht im Fokus und wird im Regelfall gar nicht erfasst [16]. Zudem erfolgt eine Beurteilung der erbrachten Leistungen häufig isoliert für die einzelnen Institutionen und nicht gemeinsam für alle an der Versorgung einer Patientengruppe Beteiligten [17]. Ein vordringliches Ziel von VBHC ist daher, den Fokus auf den „value“ der Versorgung zu richten. „Value“ ist hierbei definiert als patientenbezogenes Ergebnis („outcome“) relativ zu den aufgewandten Kosten („costs“). Patientenbezogene Outcomes werden im Konzept von VBHC über Patient-Reported Outcome Measures (PROM) und Patient-Reported Experience Measure (PREM) ermittelt [17]. Hierbei beziehen sich PROM auf die von Betroffenen empfundenen Effekte der medizinischen Intervention auf z. B. den subjektiven Gesundheitszustand, das körperliche Funktionsniveau oder die gesundheitsbezogene Lebensqualität. Dagegen messen PREM, wie Patienten die entsprechende medizinische Versorgung erleben. Dies beinhaltet z. B., ob das Anliegen, weshalb die Vorstellung im Medizinsystem erfolgte, aus Sicht der betroffenen Person adäquat adressiert wurde, aber auch wie Organisation oder Kommunikation vor Ort empfunden wurden.

Durch die Einberechnung der Kosten im Begriff „value“ wird deutlich, dass VBHC nicht ausschließlich auf eine Verbesserung des Ergebnisses für die Patienten abzielt, sondern auch einen zielführenden Einsatz der Ressourcen unter ökonomischen Gesichtspunkt fordert [16].

Im hier beschriebenen Projekt wurde VBHC auf die Gedächtnisambulanz der Uniklinik Köln angewandt. Ziel war es, einen erhöhten Nutzen für die Patienten inklusive einer erhöhten Zufriedenheit bei effizienterem Einsatz von Ressourcen zu erreichen. Hierzu wurden durch Befragung von Patienten und deren Angehörigen PREM und PROM erhoben und darauf basierend Maßnahmen entwickelt und umgesetzt, die eine Verbesserung im Sinne von VBHC darstellen sollten. Eine erneute Befragung nach erfolgter Modifikation sowie eine Analyse der eingesetzten Ressourcen erfolgten im Anschluss.

## Methoden

### Projektablauf

Das Projekt wurde vom Zentrum für Gedächtnisstörungen (ZfG) der Uniklinik Köln, dem Zentralinstitut für seelische Gesundheit (ZI) Mannheim sowie der Roche Pharma AG und der Roche Diagnostics AG entwickelt, in engem Austausch geplant und durchgeführt sowie gemeinsam analysiert und diskutiert. Die Mitarbeiter der Firmen waren wesentlich an dem Design der Studie, der Koordination und der Umsetzung sowie an der Analyse von Arbeitsabläufen und der Wirtschaftlichkeit („costs“) beteiligt. Finanziert wurde das Projekt durch die Roche Pharma AG und aus Hausmitteln der Uniklinik Köln und des ZI Mannheim. Das Projekt in Köln wurde von der Ethikkommission der Medizinischen Fakultät der Universität zu Köln positiv bewertet (Antragsnummer: 23-1291).

Im November 2023 erfolgte eine erste Befragung von 22 Patienten und deren Angehörigen. Hierauf basierend wurden veränderte Abläufe erarbeitet und in einer Pilotphase im Januar und Februar 2024 umgesetzt. In dieser Zeit wurde auch eine zweite Befragung von 21 weiteren Patienten und Angehörigen durchgeführt, um Effekte der veränderten Abläufe zu erfassen.

### Studienteilnehmer

Es wurden zu beiden Befragungszeitpunkten Patienten mit jeweils einem Angehörigen (Dyaden) in die Studie eingeschlossen, die sich erstmalig zur Diagnostik im ZfG vorstellten. Die Einschlusskriterien waren: Einwilligungsfähigkeit zur Studie, Beherrschung der deutschen Sprache und Verfügbarkeit eines Angehörigen. Die Befragungen erfolgten im Rahmen von Interviews in der Ambulanz vor dem Abschlussgespräch sowie 4 bis 6 Wochen danach telefonisch.

### Erhebung

Die Messinstrumente umfassten die Kurzversion des Patient Assessment of Chronic Illness Care Fragebogens (PACIC; [6]) zur Erhebung der subjektiven Qualität einer ambulanten Versorgung sowie die visuelle Analogskala der European Quality of Life 5 Dimensions Scale (EQ-VAS; [9]), die den subjektiv empfundenen Gesundheitszustand abbildet. Ferner wurden selbstkonzipierte prozessorientierte Fragebögen verwendet, die sich aus geschlossenen und offen formulierten Fragen zusammensetzen. Die geschlossenen Fragen bezogen sich auf subjektive Outcomes (PROM), z. B. persönlicher Nutzen der Konsultation und Erfüllung der eigenen Erwartungen, sowie auf die Abläufe (PREM), z. B. Terminkoordination. Erfasst wurden die Ergebnisse auf einer 5‑stufigen Likert-Skala (ja – eher ja – weder noch – eher nein – nein). Die offen formulierten Fragen beinhalteten u. a. Gründe für Zufriedenheit bzw. Unzufriedenheit sowie Verbesserungsvorschläge. In der zweiten Erhebung wurden ergänzende Fragen zur Evaluation der erfolgten Modifikationen gestellt. Für jeden Patienten wurde die Diagnostikdauer (Tage vom ärztlichen Erst- bis zum Abschlussgespräch), die Anzahl der notwendigen Vorstellungen und die jeweils durchgeführten Untersuchungen (Lumbalpunktion, MRT und neuropsychologische Testung [NPT]) erfasst. Zudem wurde die Anzahl und Dauer der ärztlichen und neuropsychologischen Kontakte inklusive Vor- und Nachbereitungszeiten festgehalten und zu einem individuellen Gesamtwert aufaddiert (Personalbindungszeiten). Hierbei wurde zur besseren Vergleichbarkeit vor und nach Modifikation die MR-Bildgebung nicht mit einberechnet, da diese bei der zweiten Erhebung in aller Regel bereits vor der Vorstellung im ZfG extern erfolgt war (s. unten). Die klinische Charakterisierung umfasste den Mini Mental Status Test (MMST; [5]), den Functional Activities Questionnaire (FAQ; [14]), die Geriatrische Depressionsskala (GDS, Selbstrating; [22]) und die Montgomery Asberg Depression Rating Scale (MADRS, Fremdrating; [13]), wobei die GDS in Köln und die MADRS in Mannheim als Standard eingesetzt werden. Aus Gründen der Vergleichbarkeit kamen in diesem Projekt beide Instrumente an beiden Standorten zu Einsatz.

### Datenanalyse

Für PACIC, EQ-VAS, Diagnostikdauer, Anzahl der notwendigen Vorstellungen, Personalbindungszeiten, MMST, MADRS und Alter wurden zum Vergleich vor und nach Modifikation jeweils zweiseitige *t*-Tests für unabhängige Stichproben berechnet. Die Häufigkeiten der durchgeführten Untersuchungen wurden mittels χ^2^-Tests verglichen. Für ausgewählte einzelne PROM und PREM aus den selbst konzipierten Fragebögen wurde der Exakte Test nach Fisher berechnet (vor vs. nach Modifikation). Es wurde keine Bonferroni-Korrektur angewendet, um explorativ potenzielle hypothesengenerierende Effekte aufzudecken, aber nicht konfirmative Schlussfolgerungen abzuleiten.

## Ergebnisse

Zu beiden Befragungszeitpunkten war die häufigste Ätiologie der kognitiven Defizite der Patienten die Alzheimer-Krankheit (50 % der ersten Befragung; 57,1 % der zweiten Befragung). Weitere demographische und klinische Daten sind in Tab. 1 zusammengefasst.

**Tab. 1:** Klinische und soziodemographische Daten

	Erstbefragung (vor Modifikation) 22 Dyaden	Zweitbefragung (nach Modifikation) 21 Dyaden	*p*-Wert
Patienten	*n *(%)	*n* (%)	*–*
Syndromale Diagnose			
SCD MCI Demenz	3 (13,6) 14 (63,6) 5 (22,7)	3 (14,3) 12 (57,1) 6 (28,6)	–
Ätiologie			
Alzheimer-Krankheit Depression Andere Keine Biomarkerbestimmung	11 (50,0) 1 (4,5) 3 (13,6) 7 (31,8)	12 (57,1) 3 (14,3) 1 (4,8) 5 (23,8)	–
Geschlecht (w)	11 (50,0)	14 (66,7)	–
–	*M (SD)*	*M (SD)*	
Alter (Jahre)	73,7 (6,8)	69,8 (11,4)	0,182
MMST	27,5 (2,4)	25,4 (4,9)	0,084
FAQ	5,1 (6,0)	6,2 (7,2)	0,607
MADRS	6,6 (6,0)	8,7 (6,8)	0,294
GDS	2,0 (1,8)	5,2 (3,8)	0,001
EQ VAS	71,1 (17,4)	64,9 (15,3)	0,223
PACIC	2,1 (0,4)	2,4 (0,9)	0,084
Angehörige	*n (%)*	*n (%)*	*–*
Geschlecht (w)	14 (63,6)	12 (57,1)	–
–	*M (SD)*	*M (SD)*	
Alter (Jahre)	70,1 (10,0)	60,5 (13,1)	0,010
EQ VAS	76,0 (20,0)	73,6 (20,1)	0,701
PACIC	2,2 (0,4)	2,7 (0,9)^a^	0,020

*SCD* „subjective cognitive decline“ (subjektive kognitive Störung), *MCI* „mild cognitive impairment“ (leichte kognitive Störung), *w* weiblich; *M* Mittelwert, *SD* Standardabweichung.

^a^*n* = 19 bei 2 unvollständigen PACIC-Datensätzen.

### Erstbefragung

Die Befragung zeigte grundsätzlich eine hohe Zufriedenheit mit der Diagnostik (Abb. 2). Gründe für Unzufriedenheit bzw. Verbesserungsvorschläge sind in Tab. 2 zusammengestellt. Der Punkt, der am häufigsten kritisiert und z. T. als erheblicher Belastungsfaktor angeführt wurde, war die lange Dauer des Diagnostikprozesses. Alle Patienten, die eine freie Antwort gaben (18,2 % der Befragten, *n* = 4), führten ausschließlich die Diagnostikdauer als negativ an. Eine Patientin beklagte, diese Zeit sei „furchtbar“ gewesen. Bei den Angehörigen formulierten 36,4 % der Befragten eine freie Antwort (*n* = 8). Auch hier stellte die Diagnostikdauer den Hauptkritikpunkt dar (18,2 % der Befragten, *n* = 4). Alle weiteren Verbesserungsvorschläge waren Einzelnennungen.

**Tab. 2:** Kritikpunkte und Verbesserungsvorschläge bei der ersten Befragung

	Patienten (*n* = 22)	Angehörige (*n* = 22)
Was konkret könnte man an unserem Zentrum verändern, um die Behandlung zu verbessern?/Gründe für Unzufriedenheit	Verkürzung der Diagnostikdauer *n* = 4 (18,2 %)	Verkürzung der Diagnostikdauer *n* = 4 (18,2 %)
	Keine Antwort: *n* = 18 (81,8 %)	Sonstiges* *n* = 5 (22,7 %) (Einzelnennungen)
		Keine Antwort: *n* = 14 (63,6 %)

*mehrere Punkte durch eine einzelne Person.

### Modifikationen

Die Fragen, die sich auf den subjektiv erlebten Nutzen i. S. eines PROM bezogen, zeigten bereits bei Erstbefragung eine gute Ergebnisqualität. Die Verkürzung der Diagnostikdauer als erlebter Prozess (PREM) wurde somit als Ziel einer Verbesserung im Sinne von VBHC festgelegt.

Um eine Verkürzung der Diagnostik zu erreichen und gleichzeitig die Prinzipien von VBHC zu berücksichtigen, sollte auf Diagnostikschritte verzichtet werden, die nicht unbedingt zur Ermittlung einer syndromalen und ätiologischen Diagnosen erforderlich sind. Alle Modifikationen wurden im gesamten Team des ZfG entwickelt und vor Umsetzung konsentiert.

Zunächst wurde die Dauer des ärztlichen Erstgesprächs von 60 auf 90 min verkürzt. Ferner wurde eine restriktivere Indikationsstellung für eine ausführliche NPT eingeführt, welche die Testbatterie Consortium to Establish a Registry for Alzheimer’s Disease – Neuropsychological Assessment Battery – plus (CERAD NAB+) sowie Einzeltestverfahren nach klinischen Fragestellungen umfasst. So erfolgte keine ausführliche Testung mehr, wenn durch eine relevante depressive Symptomatik valide Testergebnisse nicht wahrscheinlich waren. Zur ersten Abschätzung definierten wir hierfür einen Wert auf der GDS von ≥ 9 und auf der MADRS von > 19. Die Entscheidung gegen eine NPT wurde aber stets in Form der ärztlich-klinische Beurteilung getroffen. Die NPT konnte nach Behandlung der Depression nachgeholt werden. Auch wurden Patienten, bei denen klinisch eine Demenz mit hochgradigem Verdacht auf eine Alzheimer-Krankheit vorlag, nicht mehr ausführlich getestet (orientierende Werte: MMST < 24 und FAQ ≥ 9). Ausgenommen von beiden Regeln waren Patienten mit atypischer Symptomatik, z. B. Aphasien.

Eine weitere Modifikation betraf die Abläufe hinsichtlich der Lumbalpunktion (LP). Hier erfolgte die Aufklärung bereits bei der Erstvorstellung, sofern eine LP im Verlauf wahrscheinlich erschien, z. B. bei klinischer Demenzdiagnose. War eine NPT zur Indikationsstellung notwendig (Abgrenzung der leichten kognitiven Störung zur rein subjektiven kognitiven Verschlechterung), wurden Patienten nach dem Vorliegen der NPT-Ergebnisse angerufen, um den weiteren Ablauf in Bezug auf die mögliche Durchführung einer LP zu besprechen. Hierbei ist anzumerken, dass entsprechend der S3-Leitlinie Demenzen eine LP zur Biomarkerbestimmung bei leichter kognitiver Störung angeboten werden kann, bei rein subjektiver kognitiver Verschlechterung dagegen mangels adäquater prädiktiver Aussagekraft und fehlender Therapieoptionen nicht empfohlen wird. Die Blutabnahmen zur Bestimmung der notwendigen Gerinnungsparameter erfolgte bereits beim Erstgespräch bzw. am Tag der NPT. Damit entfiel der vorher übliche hausärztliche Termin hierfür.

**Abb. 1:**
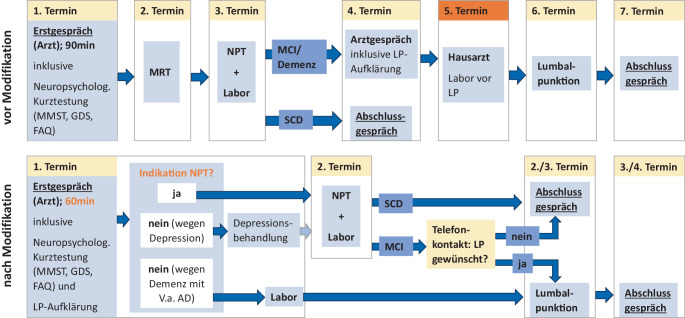
Diagnostischer Prozess vor und nach Modifikation. *MCI* „mild cognitive impairment“ (leichte kognitive Störung), *SCD* „subjective cognitive decline“ (subjektive, kognitive Störung), *AD* Alzheimer’s disease (Alzheimer-Krankheit)

Eine Hürde für die Beschleunigung der Erstdiagnostik stellten die MRT-Kapazitäten dar, welche nicht zeitnah im Rahmen der Projektdurchführung modifiziert werden konnten. Daher wurde als Voraussetzung für die Teilnahme an der Pilotphase des modifizierten Ablaufs das Vorliegen einer extern durchgeführten MRT definiert. Die diagnostischen Abläufe sind in Abb. 1 dargestellt.

### Ergebnisse der Modifikationen

Die Dauer des diagnostischen Prozesses konnte von durchschnittlich 130,2 Tagen vor Modifikation auf 21,2 Tage danach verringert werden (*p* < 0,001). Die mittlere Anzahl der Termine wurde von 4,7 auf 3,1 reduziert (*p* < 0,001). Tab. 3 zeigt, dass der Anteil der NPT nach der Modifikation mit 52,4 % niedriger als vor der Modifikation (86,4 %) war. Die Personalbindungszeiten waren nach Modifikation mit 166,4 min gegenüber 260,5 min davor reduziert (*p* < 0,001).

**Tab. 3:** Zeit und Umfang der Diagnostik

	Vor Modifikation	Nach Modifikation	
	M (SD)	M (SD)	*p-*Wert
Diagnostikdauer (Tage)	130,2 (68,9)	21,2 (12,2)	< 0,001
Anzahl der Termine	4,7 (1,2)	3,1 (0,9)	< 0,001
Personalbindungszeiten (min)	260,5 (53,1)	166,4 (51,3)	< 0,001
–	*n *(%)	*n *(%)	
Durchführung NPT	19 (86,4)	11 (52,4)	0,015
Durchführung MRT an der Uniklinik Köln^1^	12 (54,6)	2 (9,5)	0,002
Durchführung LP	14 (63,6)	13 (61,9)	1,000

^1^In der Pilotphase wurde mangels MRT-Kapazitäten die MRT im Vorfeld in radiologischen Praxen durchgeführt. Grundsätzlich ist eine Integration and der Uniklinik Köln durch entsprechende Terminplanung möglich.

**Abb. 2:**
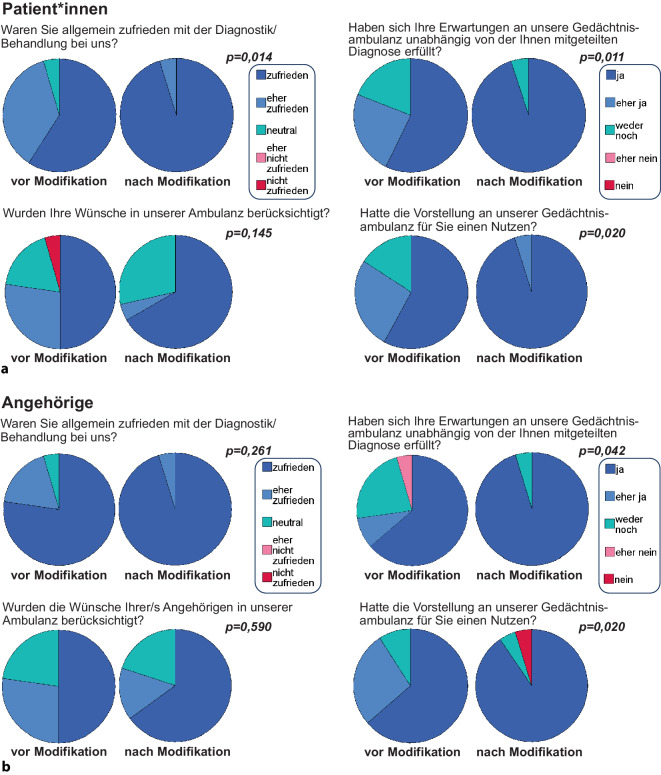
Ergebnisse der Fragebögen durch **a** die Patienten und **b** die Angehörigen zur Zufriedenheit und zum subjektiven Nutzen der Vorstellung in der Gedächtnisambulanz

**Abb. 3:**
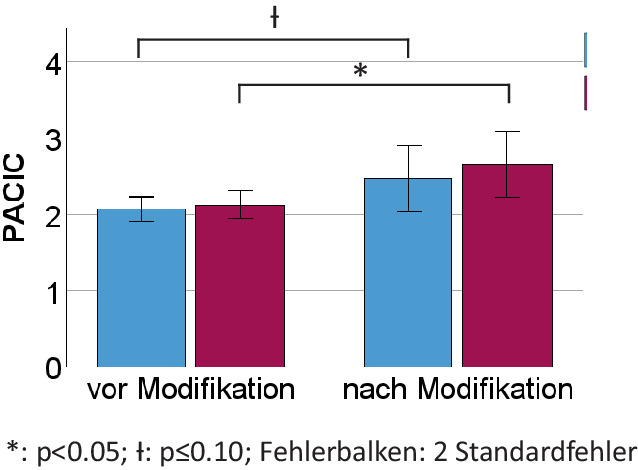
Effekte der Modifikation auf die subjektiv erlebte Qualität der Versorgung. *PACIC* Patients Assessment Chronic Illness Care (*blau* Patienten, *rot* Angehörige)

Nach der Modifikation zeigte sich in der Gruppe der Angehörigen ein besseres Ergebnis (höhere Zufriedenheit) für den PACIC (*p* = 0,02), wobei die Patienten hier nur einen Trend aufwiesen (*p* = 0,084). In den selbst erstellten Fragebögen wurde durch die Patienten eine höhere Behandlungszufriedenheit nach Modifikation angegeben (*p* = 0,014; Abb. 2). Auch die Erfüllung vorbestehender Erwartungen wurde von Patienten (*p* = 0,011) und Angehörigen (*p* = 0,042) besser bewertet. Hinsichtlich eines durch die Vorstellung generierten subjektiven Nutzens ergaben sich ebenso positivere Bewertungen in beiden Gruppen (jeweils *p* = 0,02; Abb. 3).

Bei den in der zweiten Befragung gestellten Zusatzfragen, die sich auf den verkürzten Diagnostikprozess bezogen, fanden sich folgende Ergebnisse: Die Frage „Sind Sie zufrieden mit der Vergabe der Termine?“ wurde von 94,4 % der Befragten beider Gruppen mit „ja“, von den übrigen 5,6 % mit „eher ja“ beantwortet. Auf die Frage „Was hat Sie gestört?“ formulierte keine der befragten Personen eine Antwort. Die Frage „Sind Sie zufrieden mit dem zeitlichen Ablauf der Untersuchungen und Gespräche“ wurde von 100 % der Befragten beider Gruppen mit „ja“ beantwortet. Hieran schloss sich die Frage an „Was sollte verbessert werden?“, worauf keiner der Befragten eine Antwort im Sinne eines Verbesserungsbedarfs formulierte.

## Diskussion

In dem vorliegenden Projekt wurde VBHC im Kontext einer Gedächtnisambulanz zur Analyse und Optimierung von Prozessen und Outcomes angewandt. Eine erste Befragung zeigte die grundsätzlich hohe Zufriedenheit und Erfüllung der Erwartungen der Patienten und Angehörigen in Bezug auf die Diagnostik. Kritik wurde hinsichtlich der langen Dauer bis zur Diagnose geübt. Durch Modifikation der Abläufe konnte eine Verkürzung der Gesamtzeit der Diagnostik auf im Mittel 3 Wochen erzielt werden. Auch die Anzahl der Termine und die Personalbindungszeiten („costs“) wurden reduziert. Interessanterweise führte die Modifikation aber auch zu einer Verbesserung der subjektiven Bewertung seitens der Patienten und Angehörigen („outcome“), sodass insgesamt eine messbare Steigerung des für VBHC zentralen „values“ erreicht werden konnte („value = outcome/costs“).

Eine schnelle und effiziente Diagnostik ist aufgrund der erhöhten Anforderungen an Gedächtnisambulanzen u. a. bei Einführung der neuen Antikörpertherapien von großer Bedeutung. Gleichzeitig stellt eine schnelle Diagnostik die Voraussetzung für deren Einsatz dar, da eine Zulassung nur für die Frühphase der Erkrankung bestehen wird [10]. Eine Verzögerung der Diagnosestellung könnte dazu führen, dass bestimmte Patientengruppen bei weiterer Krankheitsprogression nicht mehr für die Therapien infrage kommen [3, 10]. Ein Verpassen des richtigen Zeitpunkts wäre damit eine direkte Verletzung der Leitlinienempfehlung für eine zeitgerechte Diagnose. Wir konnten nach Modifikation bei 12 Patienten eine Alzheimer-Diagnose in einem frühen Stadium innerhalb von durchschnittlich 21,2 Tagen stellen. Die übrigen Patienten hatten andere Diagnosen, die ebenfalls in dem Zeitraum gestellt werden konnten. Eine MR-Bildgebung kann bei ausreichender Kapazität in das Zeitfenster integriert werden.

Viele Arbeiten der letzten Jahre weisen die Relevanz eines patientenzentrierten Ansatzes in der Demenzversorgung nach, auch wenn VBHC bisher in diesem Kontext kaum diskutiert wird [2, 11, 21]. Dibenedetti et al. [2] zeigen, dass sich Patienten und Angehörige von einer Behandlung der Alzheimer-Krankheit vor allem eine verbesserte Gedächtnisleistung und das Aufhalten der Krankheitsprogression wünschen. Übertragen auf den derzeitigen Forschungsstand wären diese Punkte a. e. durch eine frühzeitige und schnelle Diagnostik und den rechtzeitigen Einsatz krankheitsmodifizierender Therapien zu adressieren.

Wir haben in unserer Erhebung die subjektive Zufriedenheit der Patienten und Angehörigen erfasst. Wir haben nicht das tatsächliche Verständnis des Ergebnisses der Diagnostik und der daraus entstehenden Konsequenzen erhoben. Dieser Aspekt ist Gegenstand paralleler Forschungsprojekte [18]. Eine verständliche Aufklärung ist Grundlage für einen gemeinsamen Entscheidungsprozess über nächste Schritte (z. B. Therapie) und wesentlicher Bestandteil des patientenbezogenen Nutzens, der über die Erhebung von PROM und PREM hinausgeht. Wir haben auch nicht Effekte des modifizierten Prozesses auf die Korrektheit der Diagnose untersucht. Da aber im gleichen Umfang biomarkerbasierte Untersuchungen zur Anwendung gekommen sind, gehen wir nicht davon aus, dass sich die Qualität der Diagnostik verändert hätte.

Dies ist das erste Projekt, das das VBHC-Konzept auf Gedächtnisambulanzen in Deutschland anwendet. VBHC ist aber auch ein grundsätzlich sektorübergreifendes Konzept, was in Abstimmung mit allen Versorgern implementiert werden sollte. Neue sektorübergreifende Modelle sollten das VBHC-Konzept mitdenken, Betroffene früh einbinden und systematisch PROM und PREM erheben.

## Fazit für die Praxis

- VBHC fordert, das Verhältnis von patientenbezogenem „outcome“ zu eingesetzten Ressourcen optimal zu gestalten, um einen möglichst großen Nutzen („value“) zu generieren.
- VBHC eignet sich, um Abläufe in Gedächtnisambulanzen zu analysieren und zu verbessern, was aufgrund der neuen krankheitsmodifizierenden Therapien von besonderer Aktualität ist.
- Als zentrales Anliegen von Patienten und Angehörigen in unserer Gedächtnisambulanz wurde eine Reduktion der Diagnostikdauer identifiziert. Dies konnte in dem Projekt umgesetzt werden.
- Die Zufriedenheit der Patienten und Angehörigen wurde bei gleichzeitiger Reduktion der Personalbindungszeiten und damit der Kosten erhöht.
- Dieser Ansatz kann anderen Gedächtnisambulanzen als Modell für eine effizientere und patientenzentriertere Versorgung dienen.

## Data Availability

Patient-related raw data are not publicly available in order to protect patient privacy. Further data can be made available by contacting the corresponding author.
